# Microbial Agents as Putative Inducers of B Cell Lymphoma in Sjögren's Syndrome through an Impaired Epigenetic Control: The State-of-The-Art

**DOI:** 10.1155/2019/8567364

**Published:** 2019-01-06

**Authors:** Rossella Talotta, Piercarlo Sarzi-Puttini, Fabiola Atzeni

**Affiliations:** ^1^Department of Clinical Pharmacology and Toxicology, University of Milan, Milan, Italy; ^2^Department of Rheumatology, University Hospital ASST Fatebenefratelli Sacco, Milan, Italy; ^3^Rheumatology Unit, University of Messina, Messina, Italy

## Abstract

**Introduction:**

Understanding the mechanisms underlying the pathogenesis of Sjögren's syndrome (SS) is crucially important in order to be able to discriminate the steps that lead to B cell transformation and promptly identify the patients at risk of lymphomagenesis. The aim of this narrative review is to describe the evidence concerning the role that infections or dysbiosis plays in the epigenetic control of gene expression in SS patients and their possible involvement in B cell lymphomagenesis.

**Materials and Methods:**

We searched the PubMed and Google Scholar databases and selected a total of 92 articles published during the last 25 years that describe experimental and clinical studies of the potential associations of microbiota and epigenetic aberrations with the risk of B cell lymphoma in SS patients.

**Results and Discussion:**

The genetic background of SS patients is characterized by the hyperexpression of genes that are mainly involved in regulating the innate and adaptive immune responses and oncogenesis. In addition, salivary gland epithelial cells and lymphocytes both have an altered epigenetic background that enhances the activation of proinflammatory and survival pathways. Dysbiosis or chronic latent infections may tune the immune response and modify the cell epigenetic machinery in such a way as to give B lymphocytes an activated or transformed phenotype. It is also worth noting that transposable integrated retroelements may participate in the pathogenesis of SS and B cell lymphomagenesis by inducing DNA breaks, modulating cell gene expression, or generating aberrant transcripts that chronically stimulate the immune system.

**Conclusions:**

Microorganisms may epigenetically modify target cells and induce their transcriptome to generate an activated or transformed phenotype. The occurrence of lymphoma in more than 15% of SS patients may be the end result of a combination of genetics, epigenetics, and dysbiosis or latent infections.

## 1. Introduction

Sjögren's syndrome (SS) is a connective tissue disease that is characterized by chronic inflammation of the exocrine glands (mainly the salivary and lachrymal glands) and, in some cases, systemic involvement [1]. It can occur alone as primary SS (pSS) or accompany systemic diseases such as rheumatoid arthritis (RA) or other connective tissue diseases. Like other autoimmune diseases, its pathogenesis relies on the aberrant activation of the immune system against self-epitopes, especially in the salivary glands, a situation in which B lymphocytes play a decisive role and may undergo neoplastic transformation. In a minority of cases, in fact, SS can progress to B cell lymphoma, usually a mucosa-associated lymphoid tissue (MALT) lymphoma [2].

The initial trigger of SS is still unknown, but subjects with a permissive genetic background are more likely to develop the disease following a microbial infection. Polymorphic variants of the genes involved in the immune response have been associated with susceptibility to SS, and microorganisms such as hepatitis C virus (HCV), Epstein-Barr virus (EBV), and T lymphotropic virus type I have been considered putative inducers of the disease as well as being implicated in carcinogenesis. The mechanisms by means of which viruses can induce precancerous modifications in infected cells are still debated but may include epigenetic alterations or aberrant gene transcription following a viral genome insertion in host cell DNA. SS patients are characterized by an altered epigenetic background that may vary widely depending on the anatomical site and clinical manifestations. Epigenetic alterations (aberrant methylation, histone deacetylation, or micro-RNA expression) added to genetic predisposition may be explained on the basis of a complex crosstalk between host cells and the microbiome. It has been recently hypothesised that dysbiosis (a change in the composition of the stable commensal microbiome) is a crucial step in the pathogenesis of many autoimmune diseases as it can guide the differentiation and the activation of cells belonging to the innate and adaptive immune system. Studies of patients and experimental models of SS have described oral and gut dysbiosis in terms of its bacterial composition [3], but there are no data concerning microorganisms such as viruses, which seem to play a more important role in the pathogenesis of the disease. Latent viral infections or retroelements integrated in host DNA may impair the epigenetic machinery and prime cells to develop a proinflammatory or neoplastic phenotype.

The aim of this narrative review is to describe the current evidence concerning the role that infections or dysbiosis plays in the epigenetic control of gene expression in SS patients and their possible implication in B cell lymphomagenesis.

## 2. Materials and Methods

We searched the PubMed and Google Scholar databases for experimental and clinical studies on the potential associations of microbiota and epigenetic aberrations with the risk of B cell lymphoma in SS patients using a combination of words (Sjögren's syndrome, B cell lymphoma, epigenetics, microbiome, microbiota, virome, dysbiosis, and infections) to select the most pertinent articles. Priority was given to systematic reviews or meta-analyses and recently published original reports. Articles written in languages other than English were excluded.

## 3. Results and Discussion

Ninety-two papers published during the last 25 years were selected. The results were divided into those concerning the pathogenesis of SS, with particular focus on the epigenetic machinery that may be the means by which pathogens control the final phenotype of host cells and those concerning the role of microbial agents in determining SS and SS-associated lymphoma.

### 3.1. The Pathogenesis of Sjögren's Syndrome and Its Relationship with MALT Lymphoma

SS is a chronic autoimmune “epithelitis” whose pathogenesis seems to be triggered by an environmental agent acting on a permissive genetic background [4]. In genetically susceptible subjects, microbial agents may induce an aberrant activation of innate and adaptive immunity against mucosal epithelial cells which, far from being innocent bystanders, contribute to the inflammatory cascade by secreting cytokines and recruiting proinflammatory cells from the bloodstream.

Adaptive immunity is the most involved part of the immune response, and B lymphocytes are the hallmark of the disease. The production of autoantibodies against the ribonucleoprotein complexes Ro/SSA and La/SSB may appear several years before symptom onset, and the presence of classical infiltrates of B and T lymphocytes and plasma cells in minor salivary gland biopsies (organised in germinal centres) is mandatory for making a diagnosis and scoring disease severity [5]. Understanding the mechanisms underlying the pathogenesis of SS is crucially important in order to be able to discriminate the steps that lead to B cell transformation and promptly identify the patients at risk of lymphomagenesis.

Genome-wide association studies (GWAS) of SS patients have identified an interferon-I (IFN-I) and IFN-II signature that is possibly induced by a microbial trigger and stimulates autoimmunity [6]. The three large GWAS of SS patients and controls carried out so far have, respectively, considered Caucasian and Han Chinese populations, and subjects of European and Asian descent [7–9], and found associations between SS and signal transducer and activator of transcription 4 (STAT4), tumor necrosis factor-alpha-induced protein 3 (TNFAIP3), major histocompatibility complex (MHC) genes, and IFN regulatory factor 5 (IRF5), all of which are involved in regulating innate and adaptive immune responses. However, these studies had the biases of interracial variability, heterogeneous clinical manifestations, the nonspecific nature of the IFN signature (which is also associated with other autoimmune diseases, such as systemic lupus erythematosus (SLE) and RA) and the absence of an evaluation of patients with MALT lymphoma, which should be addressed in further studies of smaller and more homogeneous cohorts [10].

It is worth noting that the association with IRF5 highlights its presumable role in the pathogenesis of SS insofar as this mediator is involved in both viral infections and lymphomagenesis. IRF5 binds cellular DNA to its amino-terminal domain and regulates the transcription of some of the genes involved in inflammation, such as type I IFNs and other cytokines including interleukin-17 (IL-17). Some studies have shown that IRF5 may induce a permissive background to latent viral infections such as those due to EBV and favours the process of B cell transformation into malignant clones [11], whereas others have reported that IRF5 may prevent the development of hepatocellular carcinoma and that this mediator is characteristically downregulated during HCV infection [12]. Both EBV and HCV are associated with SS and may give rise to an aberrant activation of the immune system in genetically predisposed subjects. Moreover, both viruses may develop a latent phase and induce genetic changes inside host cells, thus leading to a malignant phenotype. Interestingly, it has been demonstrated that, in minor salivary glands of SS patients developing a B lymphoma, a type II IFN signature (with the production of IFN-*γ*) can overpass the type I IFN signature (represented by the IFN-*α*/*β* cascade) [13]. IFN-*γ*, secreted by T lymphocytes, natural killer cells and macrophages, plays an antimicrobial role, and its increased levels during lymphomagenesis may represent the attempt of the immune system to counteract a chronic infection.

Once the inflammatory cascade has been triggered, glandular epithelial cells undergo apoptotic and proinflammatory processes that recruit cells belonging to both innate and acquired immunity. Histological studies of salivary glands have demonstrated the presence of infiltrates of macrophages, dendritic cells, natural killer cells, and B and T lymphocytes. B lymphocytes are the main protagonists as they are responsible for cytokine release, antigen presentation, and the production of autoantibodies in a T cell-dependent or T cell-independent manner. The release of large amounts of antibodies in the circulation leads to the deposition of immune complexes and the development of systemic complications in 15% of cases [14]. At the same time, B cells from MALT may undergo an aberrant monoclonal proliferation that ends with the development of non-Hodgkin lymphoma. Some of the markers detected in minor salivary gland infiltrates and peripheral blood (including B cell-activating factor (BAFF), IL-6, IL-22, and their receptors) reflect the activation of B cells in SS, and among them, fetal liver-like tyrosine kinase 3 (FLT3) is particularly interesting as its serum concentration is directly associated with the risk of B lymphocyte transformation [15]. BAFF plays a crucial role in B lymphocyte survival and maturation, by interacting with its receptor B cell maturation antigen (BCMA), transmembrane activator and calcium modulator and cyclophilin ligand (CAML) interactor (TACI), and BAFF receptors, expressed on both B and T lymphocytes [16]. An overexpression of BAFF and its receptors in serum and tissues correlates with the risk of both autoimmune disorders and B cell-derived malignancies and the progression-free survival under chemotherapy [17–19]. Interestingly, Nezos et al. reported that polymorphic variants in *BAFF* gene were associated to an incremental risk of developing lymphoma in SS patients, with the rs9514828 T allele in the *BAFF* gene promoter conferring a high risk compared to healthy controls [20]. A similar study showed that the variant His159Tyr in the *BAFF-R* gene was associated with SS and SS-associated MALT lymphoma, especially in younger patients [21].

Immune system cells may contribute to counteract the escape of malignant clones. For example, the presence of autoreactive T cells in the autoimmune infiltrates of the minor salivary glands is usually associated with a better prognosis and a limited risk of lymphoma. T cells can be divided into cluster of differentiation 4+ (CD4+) and CD8+ cells, with the latter being more numerous in the most aggressive forms of SS that are usually associated with lymphoma risk. A multiomics study of whole-blood transcriptomes, serum proteins, and peripheral immunophenotypes in 30 Japanese SS patients and 30 controls revealed a CD8+ lymphocyte signature associated with the expression of a disintegrin and metalloprotease (ADAM), thus indicating an activated phenotype [22]. CD8+ lymphocytes are involved in the defence against intracellular pathogens such as viruses and may represent a further link between viral infection and lymphomagenesis. In this context, the increase in CD8+ lymphocytes in patients with more severe SS who are prone to develop lymphomas may represent an attempt of the immune system to prevent infected B cell proliferation.

Among the CD4+ lymphocytes, T helper 17 (Th17) cells have been associated with most severe pictures of glandular inflammation and are known to be highly pathogenic in other autoimmune diseases such as RA and spondyloarthritides [23]. They are the main producers of IL-17, a cytokine that lies at the basis of autoimmunity, but are also characterized by a high degree of plasticity as they can transdifferentiate into a Th1 phenotype depending on the cytokine milieu (such as in the presence of IL-7) [24]. Both Th17 and Th1 lymphocytes foment inflammation and favour the synthesis of other proinflammatory cytokines and chemokines by immune and epithelial cells, but Th17 lymphocytes especially promote the differentiation of B lymphocytes into plasma cells and the final production of autoantibodies. Furthermore, the production of IL-6 by B cells stimulates the differentiation of the Th17 phenotype, thus providing a loop.

Although their exact role in lymphomagenesis is still controversial, increased percentages of circulating Th17 lymphocytes have been reported in patients with B cell lymphoma and are inversely related to treatment response [25]. Accordingly, the presence of a T cell infiltrate may simultaneously strengthen immunosurveillance of transformed B cells and favour their survival and proliferation, thus playing a double-edged role in both inflammation and lymphomagenesis.

In addition to acquired immunity, the activation of innate immune system cells may further amplify local and systemic inflammation and provide a direct link between infections and autoimmunity or lymphomagenesis. Toll-like receptors (TLRs) are abundantly expressed in cells belonging to the innate immune system and may cross-recognize molecules from damaged cells, including cancer cells (damage-associated molecular patterns (DAMPs)) and pathogens (pathogen-associated molecular patterns (PAMPs)). In SS patients, the stimulation of TLR2 and TLR4 on the plasma membrane of peripheral blood mononuclear cells (PBMCs) by bacterial compounds such as lipopolysaccharide (LPS) induces the production of IL-17, whose effects on B lymphocytes have been discussed above. Salivary epithelial gland cells recognize viral genome by means of intracellular TLR3 and induce the expression of CD40, intercellular adhesion molecule 1 (ICAM-1), MHC class I, and BAFF, which promote the activation and proliferation of B cells [26]. The activation of the innate immune system via the TLR pathway has also been found in proteomic studies of SS patients' saliva, which aberrantly expresses molecules such as S100 family proteins [27]. Interestingly, many of these molecules are produced in response to infections or alterations in commensal flora and a changed salivary proteomic profile has been observed in a SS patient developing a MALT lymphoma [28], thus underlining the possibility that microbial insults may give rise to inflammation and neoplastic transformation in SS patients. Accordingly, the use of proteomics in the search for salivary markers may be a useful means of distinguishing healthy and SS subjects and allowing the early identification of patients at risk of developing a MALT lymphoma.

### 3.2. Epigenetics

A number of studies have demonstrated that patients with SS and other autoimmune diseases have an impaired epigenetic profile consisting of aberrant DNA methylation, histone deacetylation, and/or the production of noncoding micro-RNA [29]. Miceli-Richard et al. analysed the methylation of PBMCs from 26 SS patients and 22 controls using Infinium Human Methylation 450 K BeadChips [30] and found significant differences in methylation patterns between patients and controls and between CD19+ B lymphocytes and CD4+ T lymphocytes, with 391 epigenetically modulated genes encoding proinflammatory proteins. This modulation was associated with the EULAR Sjögren's syndrome disease activity index (ESSDAI) and the production of anti-Ro/SSA and anti-La/SSB autoantibodies. The authors found 22 differentially methylated genes in 5% of the SS patients developing a B cell lymphoma, including tumor necrosis factor (TNF), TNF receptor superfamily member 10A (TNFRSF10A), IFN-γ, IFN-α, TLR3, and TLR7. The different expression of genes related to intracellular pathogen infections (TLRs, the IFN pathway) in transformed B lymphocytes reinforces the hypothesis of a crosslink between infections, epigenetics, and lymphomagenesis. It is interesting to note that no change in global methylation was detected in the interspersed DNA *Alu* sequences or long interspersed elements (LINEs), which are usually sites of retroviral integration, whereas up to 40% involved 5′—C—phosphate—G—3′ (CpG) islands that are usually associated with the promoter region. Altorok et al. described different patterns of methylation in peripheral CD4+ T cells in 11 Caucasian patients with pSS and controls [31]. They found a total of 426 differently methylated genes, most of which were hypomethylated and consequently overexpressed. Among these, lymphotoxin-α (LTA) is particularly interesting because of its role in the activation of innate and acquired immune responses, lymphoid organogenesis, and antiviral responses [32]. Therefore, according to these studies, both T and B lymphocytes from SS patients are characterized by an aberrant epigenetic control of genes involved in microbial defence, inflammation, and cell survival, finally converging to an activated phenotype.

Other authors found nearly 40% homology in the methylation patterns of genes taken from salivary gland epithelial cells and peripheral B and T lymphocytes obtained from 16 SS patients and 4 controls [33] but demonstrated a different methylation of genes related to the calcium pathway and wingless-type MMTV integration site family (Wnt) signalling, which are known to be associated with oncogenesis and cell survival. The calcium pathway is involved in oxidative stress and the unfolded protein response (UPR) due to rough endoplasmic reticulum (RER) stress. It has been demonstrated that salivary gland epithelial cells have a phenotype characterized by RER stress and altered mucin protein secretion, which may be related to unfolded proteins retained in the RER that may activate innate responses through the nuclear factor kappa-light-chain-enhancer of activated B cells (NF-*κ*B) and activator protein-1 (AP-1) pathway and the NLR family pyrin domain-containing 3 (NLRP3) inflammasome [34]. Inflammasomes are multiproteic complexes elicited by the recognition of pathogen domains that may derive from microorganisms or cellular debris and cooperate with the TLR pathway in the innate immune response [35, 36]. The activation of the NLPR3 inflammasome in epithelial cells of SS patients has been demonstrated in a histological study of five SS patients with concomitant MALT lymphoma, and the P2X7-IL-1-beta-IL-18 signature on salivary glands (but not on CD20+-infiltrating B lymphocytes) seems to correlate directly with the risk of lymphomagenesis [37].

Finally, other authors have described the hypomethylation of the genes involved in the IFN pathway in whole blood, CD19+ B lymphocytes, and minor salivary gland biopsies of pSS patients [38]. It is not clear how epigenetic aberrations in epithelial salivary glands dictate the activation and transformation of B lymphocytes into malignant cells, but it is possibly because of a paracrine mechanism.

Interestingly, all of these studies agree on the hyperexpression of interferon-induced protein 44-like (IFI44L) in the B and T lymphocytes and whole blood of SS patients: this protein prevents the replication of various human viruses, including HCV, yellow fever virus, West Nile virus, chikungunya virus, and human immunodeficiency virus-1 [39], thus suggesting that the altered methylation has a viral etiology.

The methylation of CpG islands is the main mechanism underlying the inactivation of the X chromosome in females. As SS is prevalent in women, impaired methylation of the X chromosome (which contains the largest number of genes coding for molecules related to the immune response, including TLR7) has been suggested and demonstrated in salivary gland epithelial cells of SS patients [40]. The X chromosome inactivation process depends on LINE1 sequences, and aberrant epigenetic changes may occur in the case of unusual insertions or transpositions of a foreign genomic material. Methylene tetrahydrofolate reductase (MTHFR) is the rate-limiting enzyme in the methylation cycle, and polymorphic variants in its gene are associated to DNA damage and increased risk of autoimmune and lymphoproliferative disorders. According to a recent study, SS patients with a non-MALT lymphoma had increased expression of the *MTHFR* c.677C>T T allele and a reduced expression of the *MTHFR* 1298 A>C C allele compared to healthy controls, whereas no significant differences in *MTHFR* gene polymorphisms emerged when SS patients with MALT lymphoma were considered [41]. These genotypes were associated to reduced DNA methylation and genomic instability, thus providing an explanation to mutagenesis in non-MALT lymphoma, which may have a distinct epigenetic pathway from MALT lymphoma.

In addition to DNA methylation, aberrant micro-RNA expression has also been described in the pathogenesis of SS [42]. Micro-RNAs are small noncoding RNA filaments that are 19-23 nucleotides in length and control gene expression mainly by pairing the 3′untraslated region (3′UTR) of target mRNAs, although other mechanisms of control have also been described [43–45]. Aberrant micro-RNA expression may be related to an exaggerated inflammatory response, and studies of PBMCs and salivary gland epithelial cells have detected altered micro-RNA 146a/b, the 17-92 cluster, and 181a expression in SS patients [46]. It has been reported that micro-RNA 146a is upregulated in PBMCs and salivary glands of SS patients [47, 48], that micro-RNA 181a and 16 are downregulated in salivary glands [49], and that cluster 17-92 is upregulated in the lymphocytes of patients with lymphoproliferative disorders and downregulated in patients with SS [50, 51]. The expression of micro-RNAs is tissue-related and may be induced by infectious stimuli: for example, infections due to *Helicobacter pylori* seem to modulate the expression of some micro-RNAs controlling cell proliferation and survival. Floch et al. have demonstrated in a gastric MALT lymphoma mouse model that chronic *Helicobacter pylori* infection may induce the overexpression of micro-RNAs 21a, 135b, 142a, 150, and 155, which prevents the transcription of the gene coding for the proapoptotic protein tumor protein P53-inducible nuclear protein 1 (TP53INP1) [52]. Some of these micro-RNAs control the production of antibodies (including autoantibodies) [53], and others may belong to viruses infecting host cells as in the case of EBV infection [54]. Viral micro-RNAs such as EBV micro-RNA 181a may be overexpressed in infected cells and favour cell survival and proliferation, thus paving the way for the development of proliferative disease. The expression of micro-RNA 155, which is involved in B cell growth, is enhanced in lymphomas, and its overexpression may be driven by EBV infection through two EBV-encoded proteins: latent membrane protein-1 (LMP1) and EB nuclear antigen-2 (EBNA2) [55]. Microbial infections may therefore modulate cell fate by means of epigenetic control and lead to the final development of a proinflammatory or neoplastic phenotype.

### 3.3. Dysbiosis and Infections

The human microbiome, which consists of 100 trillion commensal bacteria, protozoa, fungi, and viruses living inside and on the human body, is now widely considered an independent organ *per se* that is capable of interacting with host cells and guiding the differentiation of the immune system [56], and this has led to growing interest in investigating the role of alterations in microbiome composition (dysbiosis) in the pathogenesis of autoimmune and neoplastic diseases. Commensal microorganisms compete with pathogens for substrates and do not usually induce an immune response because of the physical barriers that confine them to specific anatomical sites and impede their entry in the bloodstream and the active immunosurveillance exerted by resident and circulating immune cells. The gut harbours the majority of commensal microorganisms, and a variation in gut flora has been associated with an imbalance in the Th17/T regulator cell ratio [57, 58]. Although the microbiome is quite stable during the course of life, changes in its composition may be dictated by various factors, including antibiotic therapies and diet, and the combination of genetically mutated resident microorganisms and a favourable genetic background in host cells may alter this balance and induce dysbiosis. This condition, which is characterized by the emergence of pathogenic *phyla*, lies at the basis of many autoimmune diseases as it may elicit sterile inflammation and prime the innate and acquired immune system [35]. Pathogen derivates (proteins or nucleic acids) are capable of interacting with the proteins in nucleotide-binding domain-like receptors (NLRs), absent in melanoma 2-like receptors (ALRs), and pyrin, and this activates the NLPR3 inflammasome [59].

Various studies of SS patients and animal models have demonstrated dysbiosis in the gut and oral mucosa [3]. Patients with SS may develop antibodies against the highly conserved microbial proteins of *Staphylococcus aureus* and *Escherichia coli*, and as a result of molecular mimicry, these can cross-react against mitochondrial self-antigens, which may explain their symptoms of fatigue [60]. A continuous attempt to defend the human body against pathogens may also explain the aberrant activation of B lymphocytes and plasma cells and the subsequent production of antimicrobial and autoantibodies characterizing SS. Studies of microbial balance in the oral mucosa of SS patients have led to discordant findings: for example, the risk of periodontitis does not seem to be higher in SS than in controls [61], although SS mouse models and patients show a specific microbial gut signature with the prevalence of *Enterobacteriaceae* over *Bifidobacteriaceae* that is finally responsible for autoimmunity [62].

Viral infections (especially those due to EBV and hepatitis viruses) are quite frequent in SS patients. These viruses share the ability to integrate themselves into the host cell genome and use its transcriptional machinery to change latent to lytic infection [63, 64]. This is ultimately responsible for DNA breaks and aberrant gene transcription. EBV infection has been associated with the risk of developing a lymphoma. The virus infects hematopoietic and epithelial cells and escapes clearance by the immune system by expressing different antigens at different stages of the infection. During latency, it expresses six EBNAs, which immortalise B lymphocytes by preventing the activation of the p53 pathway or enhancing kinase activity [65]. In comparison with controls, a Colombian study of 82 SS patients found a significant increase in anti-EBV early antigen (EBVEA) immunoglobulin G (IgG), which is indicative of a lytic infection and directly correlates with anti-Ro/SSA/SSA52 and anti-La/SSB antibody titres and inversely with anti-RNP-68 titres [66]. EBVEA is structurally and functionally similar to protooncogene B cell lymphoma 2 (bcl-2), which is involved in cell proliferation and survival and carcinogenesis [67]. EBV-infected epithelial cells and B lymphocytes may therefore express different viral antigens depending on the phase of the infection, and the relative changes in transcriptomes may underlie the emergence of a neoplastic phenotype.

Retroviruses can insert themselves into host cell DNA, modulate the transcription of cellular genes, or generate aberrant transcripts that activate the innate immune response. Infections due to human immunodeficiency virus-1 (HIV-1) and type 1 human T lymphotropic virus can induce the onset of sicca syndrome, and studies of animal models and patients with SS have shown the presence of retroviral antigens or genomic sequences in salivary glands as well as serum antibodies reacting against retroviral Gag proteins [68].

In addition to viral infections, the human virome (which mainly consists of bacteriophages) may induce bacterial dysbiosis, which is associated with the development of autoimmune diseases such as inflammatory bowel diseases or type I diabetes [69]. However, there are still no data available concerning the role played by commensal virome in the pathogenesis of SS.

The cellular genome also contains retroelements or latent viral insertions that may activate inflammasomes [70]. These elements, which account for nearly 50% of the human genome, are movable (transposons) and roughly fall into two groups: those with long terminal repeats (LTRs) containing genes for reverse transcriptase and those without (including LINEs and short interspersed elements (SINEs) such as *Alu*). Most cannot retrotranscribe but they can affect the transcription of genes adjacent to their insertional site. Cells are usually capable of distinguishing exogenous nucleic acids and their own genome on the basis of the molecular structure (length or nucleotide composition), but there is some evidence indicating that endogenous *Alu* retroelements can form complexes with cellular ribonucleoproteins such as Ro60 and activate TLR pathways [71] and SINE RNAs can directly induce the NLRP3 inflammasome [72]. Interestingly, NLRP3 is activated by 5′ uncapped nucleic acids that may belong to pathogens such as viruses or damaged cells (e.g., cancer cells) and nucleic acids and ribonucleoproteins can activate AIM2 inflammasome and contribute to the progression of SLE [73]. It has been shown that NLRP3 and AIM2 activation is increased in the PBMCs and salivary gland-infiltrating macrophages of SS patients [74, 75] and that activation of the inflammasome in SS epithelial cells is directly associated with the likelihood of developing a B cell lymphoma [37]. According to the results of a fascinating ex vivo study on minor salivary gland biopsies of SS patients, it is likely that an aberrant methylation of LINEs1 inside epithelial cells and the concomitant expression of open reading frame 1/p40 protein would induce their recognition by TLR7 and TLR8, activate the NF-*κ*B pathway, and generate a type I IFN signature [76]. The reactivation of L1 retroelements relies on different patterns of methylation, and it has been shown that minor salivary gland tissues from SS patients at high risk of lymphoma have an increased methylation in L1 elements and a concomitantly reduced expression of some methylating enzymes, including DNA methyltransferases 3B and 1 and methyl CpG-binding protein 2, compared to those from patients at low risk [77]. However, there is still a lack of studies of retroelements in SS patients or experimental models and there is still uncertainty concerning their role in triggering the disease or its complications, including B cell lymphoma.

### 3.4. Infections, Dysbiosis, and Lymphomagenesis in Patients with Sjögren's Syndrome

It is still unclear whether the epigenetic changes induced by chronic latent infections can trigger the development of SS and SS-associated lymphomas in genetically predisposed subjects. Pathogenic and sterile infections may both contribute to the disease by chronically stimulating B lymphocyte clones. Patients with pSS produce polyclonal antibodies and have increased levels of C19^dim^CD138+ plasmablasts following H1N1 influenza vaccination as a result of hyperactive B cells [78]. Like other autoimmune diseases, in SS, the primary site of infection is often unknown and it is possible that lymphomas may be triggered by a previous infection that epigenetically primes the immune system and whose final repercussions affect districts that may be far from the original site of infection. In support of this hypothesis, experimental Fas-deficient C57Bl/6-lpr/lpr mice models have shown that infection with murine cytomegalovirus is followed by the development of salivary gland infiltrates with undetectable virus [79]. In addition, multiple consecutive infections may epigenetically modify target cells to develop a final cancerous or proinflammatory phenotype. The risk of B cell lymphoma increases with the duration of SS (approximately 4% in the first five years of disease and up to 18% after 20 years) and in patients with lymphadenopathy, parotid enlargement, palpable purpura, cryoglobulinemia, and low serum C4 levels [2].

The most frequent SS-associated lymphoma is MALT lymphoma [80], but SS patients may develop other lymphoproliferative disorders such as follicular lymphoma or diffuse large B cell lymphoma. MALT lymphoma develops from marginal zone B cells, which probably influence the severity of the disease in terms of autoantibody production, histological lesions, and symptoms [81]. Depending on their anatomical site, marginal zone lymphomas can be divided into classical MALT lymphomas (which arise from lymphoid follicles associated with mucosa), splenic marginal zone lymphomas, and nodal marginal zone lymphomas. Genetic and epigenetic changes have been found in each form, including chromosomal trisomies, single-nucleotide polymorphisms, and methylation changes [82]. All of these phenomena lead to the aberrant activation of NF-*κ*B, which promotes the survival and activation of B cells. It is worth noting that the TNFAIP3 somatic mutation is found in approximately 28% of all MALT lymphomas and the dysregulation of this gene has also been associated with SS in GWAS [7–9, 83]. Of note, in a study of human coronavirus 229E- (HCoV-229E-) infected A549 and HuH7 cells, TNFAIP3 was typically upregulated in order to guarantee efficient viral replication [84].

An interesting experiment involving homozygous 564Igi^+/+^ mice carrying insertions at the heavy and light chains of autoantibody 564, which reacts against nucleic acids and nucleoproteins, has shown that lymphomagenesis may be related to autoimmunity and that both may be prevented by the antibiotic treatment of gut microbiota [85]. More specifically, it demonstrated that IL-21 may be simultaneously responsible for lymphocytic transformation, plasmablast activation, and the final production of autoantibodies. These mice were prone to develop multidistrict infections, but when treated with antibiotic supplements acting on intestinal flora, they showed no lymphatic expansion, risk of lymphoma, hypergammaglobulinemia, or autoimmune diseases. Consequently, the authors argued that changes in microbiota may guide the differentiation of plasmablasts via a TLR7/IL-21 pathway, thus leading to lymphoproliferative disorders or autoimmunity and impairing the adaptive response against pathogenic microorganisms. Nevertheless, spleen sections of mutant and wild-type mice did not show any changes in marginal zone architecture, the starting point of MALT lymphomas in SS patients. This may be explained by differences in the anatomical sites of emergence of malignant B cells, which is usually the surrounding oral mucosa in SS patients.

There is much evidence that MALT lymphomas may follow infections due to *Chlamydophila psittaci*, *Campylobacter jejuni*, and *Helicobacter pylori* and they can be sometimes eradicated by antibiotic therapy in the earlier phases of development [86]. Bacterial or viral infections may generate autoimmunity by means of a mechanism of molecular mimicry and the polyclonal activation of B lymphocytes. A high degree of inflammation, mirrored by more severe clinical manifestations and laboratory findings, is associated with the risk of lymphomagenesis. Higher anti-Ro/SSA and anti-La/SSB autoantibody titres or autoantibody positivity is associated with an increased risk of B cell lymphoma in SS patients and with the *de novo* onset or relapse of cancer in SLE patients [87].

A fascinating alternative to chronic infectious stimulation of B cells is the possibility of alterations in gene transcription due to transposable retroviral elements or latent virus infections. Latent EBV transcripts (including EBNAs and LMPs) epigenetically regulate the transcription of viral oncoproteins by means of CpG methylation, histone deacetylation, micro-RNA pairing, or super enhancer activation [88], and the same mechanisms may act to immortalise host cells and lead to the acquisition of a transformed phenotype. In addition, EBV expresses two noncoding RNAs (EBER-1 and EBER-2), both of which have been implicated in B cell lymphomagenesis.

Human endogen retroviruses (HERVs) represent the “ghosts” of long-latent viral infections whose fully integrated genome accounts for 8% of human DNA. It has been hypothesised that HERVs favour disease onset in a pathogenic model of multiple sclerosis (MS) by inducing or repressing the transcription of the MS causative gene in *cis*, by transcribing the HERV proteins responsible for the formation of autoantigens or inducing RER stress, and, finally, by cooperating with infections due to common viruses such as EBV or influenza virus [89]. HERVs are usually untrascribed, but the results of a recent RNA sequencing experiment analysing the behaviour of HERVs in murine and human B cells in various pathological contexts are intriguing [90]. The authors examined B cell HERV transcriptome under infectious, autoimmune, and neoplastic conditions and found that LINE transcription was slightly enhanced in sepsis, LTR transcription increased in response to IFN (and therefore autoimmune diseases), and LTRs and LINEs were most highly expressed in B cell lymphoma. Interestingly, the HERVs expressed in transformed B cells did not have the same sequences as those expressed in B cells activated by an inflammatory stimulus. Mouse B cell lymphomas were characterized by the increased expression of the provirus *Emv2*, which may have acquired infectiousness following the recombination of genomic strands.

Cancer cells can escape immunosurveillance as a result of T cell exhaustion and the increased expression of the immune checkpoints that prevent immune cell activation (programmed death (PD-1) or cytotoxic T lymphocyte antigen 4 (CTLA4)). Cancer patients treated with the new antitumoral agents known as checkpoint inhibitors may develop autoimmune symptoms. SS has been reported in four patients treated with ipilimumab and nivolumab, although it is interesting to note that none of the patients was positive for anti-Ro/SSA antibodies, thus suggesting a different pathogenesis [91]. Latent infections may epigenetically control immune checkpoints as it has been demonstrated that EBV can induce the expression of PD-1 by downregulating micro-RNA 34 in B cell lymphoma [92].

**Figure 1:**
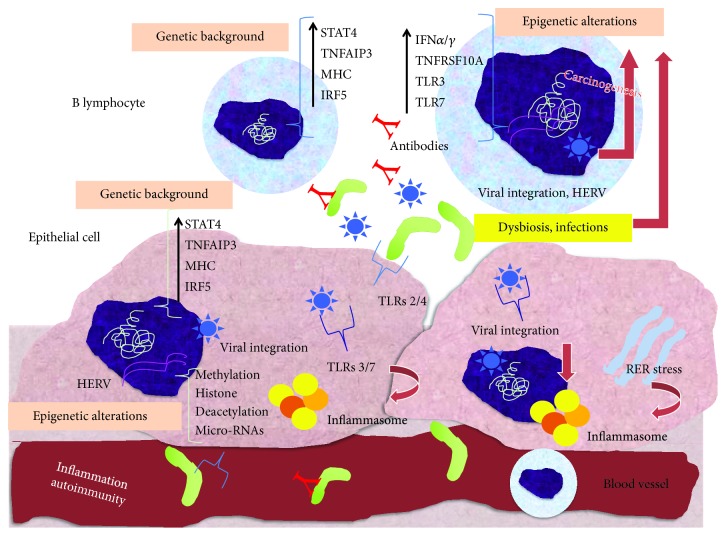
Pathogenesis of Sjögren's syndrome and associated B cell lymphoma. On the basis of a favourable genetic background, represented by the hyperexpression of genes related to the inflammatory cascade (STAT4, TNFAIP3, MHC, and IRF5) shared by both B lymphocytes and salivary gland epithelial cells, infections or dysbiosis may modify the cell epigenetic machinery and consequently control gene expression, favouring inflammation, autoimmunity, and lymphomagenesis. Particularly, the epigenetic induction of the transcription of genes associated to microbial recognition (e.g., TLR3 and TLR7) and IFN-I signature, by means of aberrant methylation, histone deacetylation, and micro-RNA expression, may further promote the hyperactivation of B cells in response to foreign or endogenous epitopes, generated by bacteria, viruses, and endogenous retroelements. In addition, RER stress and integrated retroelements may favour the activation of inflammasomes and cause cell DNA rearrangements, which represent crucial steps for chronic B cell activation and transformation. These steps finally converge to the hyperproduction of autoantibodies cross-reacting with antigens locally or systemically, inflammation and destruction of glandular tissues, and B cell transformation into malignant clones. STAT4: signal transducer and activator of transcription 4; TNFAIP3: tumor necrosis factor-alpha-induced protein 3; MHC: major histocompatibility complex; IRF5: interferon-regulatory factor 5; IFN: interferon; TNFRSF10: tumor necrosis factor receptor superfamily member 10A; TLR: Toll-like receptors; HERV: human endogen retroviruses; RER: rough endoplasmic reticulum.

Figure 1 summarises the presumable mechanisms underlying the pathogenesis of SS and SS-related B cell lymphoma.

## 4. Conclusions

Like that of other autoimmune diseases, the pathogenesis of SS is due to interactions between a favourable genetic background and an environmental trigger such as infections or dysbiosis. Microorganisms may induce epigenetic modifications in target cells and tune their transcriptome to generate an activated or transformed phenotype. The establishment of lymphoma in more than 15% of SS patients may be the end result of a combination of genetics, epigenetics, dysbiosis, or latent infections. Interestingly, there is increasing evidence that retroelements integrated in cell DNA participate in the process of lymphomagenesis and autoimmunity, perhaps by modulating the expression of key genes; however, there is still a lack of studies specifically investigating their precise role in the pathogenesis of SS and SS-associated B cell lymphoma.
